# Pan-cancer analysis of RNA expression of ANGIOTENSIN-I-CONVERTING ENZYME 2 reveals high variability and possible impact on COVID-19 clinical outcomes

**DOI:** 10.1038/s41598-021-84731-7

**Published:** 2021-03-11

**Authors:** Andrew Elliott, Michelle Saul, Jia Zeng, John L. Marshall, Edward S. Kim, Misako Nagasaka, Heinz-Josef Lenz, Lee Schwartzberg, David Spetzler, Jim Abraham, Joanne Xiu, Phillip Stafford, W. Michael Korn

**Affiliations:** 1 Caris Life Sciences, 4610 South 44th Place, Phoenix, AZ 85040 USA; 2 Ruesch Center for the Cure of Gastrointestinal Cancers, Lombardi Comprehensive Cancer Center, Georgetown University Medical Center, Washington, DC USA; 3 Levine Cancer Institute, Atrium Health, Charlotte, NC USA; 4 Department of Oncology, Karmanos Cancer Institute, Wayne State University, Detroit, MI USA; 5 University of Southern California, Keck School of Medicine, Norris Comprehensive Cancer Center, Los Angeles, CA USA; 6 Medical Oncology, West Cancer Center, 9745 Wolf River Blvd, Germantown, TN USA; 7 Division of Hematology/Oncology, Department of Medicine, University of California San Francisco, San Francisco, CA USA

**Keywords:** Cancer genomics, Virology

## Abstract

Patients with cancer demonstrate particularly poor outcomes from COVID-19. To provide information essential for understanding the biologic underpinnings of this association, we analyzed whole-transcriptome RNA expression data obtained from a large cohort of cancer patients to characterize expression of ACE2, TMPRSS2, and other proteases that are involved in viral attachment to and entry into target cells. We find substantial variability of expression of these factors across tumor types and identify subpopulations expressing ACE2 at very high levels. In some tumor types, especially in gastrointestinal cancers, expression of ACE2 and TMPRSS2 is highly correlated. Furthermore, we found infiltration with T-cell and natural killer (NK) cell infiltration to be particularly pronounced in ACE2-high tumors. These findings suggest that subsets of cancer patients exist with gene expression profiles that may be associated with heightened susceptibility to SARS-CoV-2 infection, in whom malignant tumors function as viral reservoir and possibly promote the frequently detrimental hyper-immune response in patients infected with this virus.

## Introduction

The new severe acute respiratory syndrome coronavirus, SARS-CoV-2, has led to a devastating pandemic affecting large segments of the global population with 70,111,812 confirmed cases and at least 1,591,595 deaths as of December 11, 2020^1^. The disease resulting from SARS-CoV-2 infection, COVID-19, shows particularly severe courses in men and the elderly, a relationship that is further aggravated by comorbidities^2^. Malignant diseases are comorbidities of interest in this context, as cancer patients, whose frequent treatment with immune-suppressive drugs might add to their vulnerability, have demonstrated particularly severe courses of the disease^3,4^. Despite the similarities of SARS-CoV-2 to other coronaviruses^5–7^, the biologic underpinnings of increased COVID-19 morbidity remain unclear.

SARS-CoV-2 utilizes the ANGIOTENSIN-I-CONVERTING ENZYME 2 (ACE2) cell surface receptor for host cell entry^8^. Interaction with ACE2 is mediated by the viral spike protein, which undergoes proteolytic pre-activation, or priming, by cellular proteases TMPRSS2 and cathepsins (CTSB/CTSL)^5^. The SARS-CoV-2 spike protein also contains a novel cleavage site for the proprotein convertase FURIN that is absent in SARS-CoV^6^. FURIN cleavage of the SARS-CoV-2 can preactivate the spike protein for cell entry, and TMPRSS2 and CTSB/CTSL have cumulative effects with FURIN on SARS-CoV-2 entry^9^. Interestingly, increased expression of ACE2 and FURIN have also been observed in oral epithelial cells^10^.

Non-small cell lung cancer (NSCLC) patients infected by SARS-CoV-2 have a higher risk of severe symptoms and higher mortality rate than patients without^11,12^. As ACE2 expression is related to the severe acute respiratory syndromes induced by SARS-CoV via mediating the production of cytokines and the resultant adaptive immune response^13,14^, we examined transcriptomic markers of immune and stromal cell populations in the tumor microenvironment (TME) of NSCLC specimens^15^. Understanding how the tumor microenvironment changes in NSCLC as ACE2 expression increases may provide additional insight into the associated immune system deregulation that may underlie the severe comorbidities observed in NSCLC patients and may provide additional insight into an effective treatment for those suffering from COVID-19.

Initial data suggest that some cancers express high levels of ACE2^16,17^. In such cases, malignant masses could function as viral reservoirs, similar to observations related to other viruses, thus sustaining and amplifying SARS-CoV-2 infection in patients with cancer^18,19^. Additionally, increased co-expression of cellular proteases with ACE2 may further identify subsets of cancer patients with increased vulnerability to infection^20,21^. Here, we endeavored to systematically characterize ACE2 and key cellular protease gene expression in normal and malignant tissues, taking advantage of a large database of tumors profiled by whole-transcriptome sequencing (WTS). We hypothesized that ACE2 and protease gene expression would vary across tumor types, with high expression presumed to associate with increased risk of infection and severe course of disease. We found that ACE2 expression varies and correlates with expression of proteases in a tumor type-specific manner, which may underlie the severe disease progression observed in subsets of patients with cancer.

## Methods

As part of routine comprehensive molecular profiling of cancer samples at the Caris Life Sciences laboratory, a NextGen RNA sequencing panel was utilized that uses a hybrid bait-capture method to pull down RNA transcripts and sequence them. FFPE biopsies are obtained by the lab, sliced to 0.7 mm by microtome, and mounted on slides. Tumor tissue is identified by pathologists and microdissected. Tumor percentage is estimated. RNA preparation and cDNA reverse transcription follows manufacturer’s guidelines (Qiagen). Library prep is standard for Illumina NextSeq two-color sequencing. RNA preparation follows manufacturer’s guidelines. RNA transcripts are pulled down by the Agilent V7 Whole Exome bait panel. The bait panel is the Agilent SureSelect Whole Human Exome hg19 V7. For their whole-exome panel they avoided pseudogenes and highly redundant genes, resulting in 22,192 annotated transcripts. The RNA-Seq assay sequences the captured exonic mRNAs and assembles them onto the hg19 genome scaffold by STAR Aligner^22^.

The Illumina NovaSeq 6500 was used to sequence the whole transcriptome from patients to an average of 30 M paired end reads. Raw data was demultiplexed by Illumina Dragen BioIT accelerator, trimmed, counted, PCR-duplicates removed and aligned to human reference genome hg19 by STAR aligner^22^. NGS RNA sequencing captures 22,192 exonic regions. For transcription counting, transcripts per million molecules (TPM) was used (Salmon expression pipeline^23^). TPM expression data were pulled from a relational database containing retrospective clinical sample information. In addition to the large cohort of tumor samples tested, a small panel of patients where non-tumor tissue was extracted and tested was examined for ACE2 expression. Pathway analysis was performed using KEGG pathway colored mapping on Z-scores of fold change between groups^24^. All analyses were performed on deidentified, retrospective cases. The study protocol was reviewed and approved as exempt from full IRB review by the Western Institutional Review Board (WIRB) and need of informed consent has been waived by WIRB. The study was conducted in accordance with the Declaration of Helsinki and adhered to Good Clinical Practice guidelines. Expression data were used to build data distributions, correlate cohorts, and examine gene expression outliers, and perform statistical tests. Statistical analyses were performed using R version 3.6.1^25^. Pearson’s product moment correlations were calculated and associated p-values generated via the cor.test function in the stats package.

## Results

### Patient and tumor characteristics and correlations with ACE2 expression

A total of 38,628 tumors specimens, representing 36 tumors types, that underwent comprehensive molecular profiling at Caris Life Sciences between 2019 and 2020 were retrospectively reviewed. Molecular profiles of adjacent normal (non-cancerous) tissue from 127 tumor specimens, representing 23 tumor types (Supplementary Table 1), were reviewed as a composite control. Key demographic characteristics of these cohorts are shown in Table 1.

**Table 1:** ACE2 expression associated with patient demographics of normal control and cancer cohorts.

Characteristic	ACE2 Expression (TPM)									
	Cancer					Normal				
	N samples	Mean	Median	Range	p-value	N samples	Mean	Median	Range	p-value
**Gender**										
Female	22493	4.8	0.74491	0.006422–3385.94	< 0.0001	75	3.53	0.73799	0–102.946	0.02
Male	16135	6	0.88101	0.007438–883.672		52	22	1.58763	0–409.937	
**Age Group (years)**										
< 65	19944	5.8	0.804511	0.006422–3385.94	< 0.0001	70	8.62	1.23194	0–202.582	0.24
≥ 65	18684	4.76	0.790774	0.007306–1495.36		57	14.1	0.94495	0–409.937	
**Smoking status**										
Current smoker	1039	3.49	0.784241	0.008451–204.511	0.42	3	6.5	0.56718	0.125167–18.794	0.79
Lifelong non-smoker	322	3.38	0.990115	0.008251–105.225		0	NA	NA	NA	
Light smoker (<15 packs/yr)	2479	4.13	0.900375	0.008565–549.111		6	69.1	1.00519	0.091297–409.937	
Not reported	34788	5.45	0.786538	0.006422–3385.94		118	8.25	1.02695	0–208.806	

P-values were calculated using the Kruskal–Wallis rank sum test for numeric variables and Pearson’s Chi-squared test for categorical variables.

Overall, average ACE2 expression was significantly increased in patients under 65 years compared to patients 65 years and older (5.8 vs 4.8 TPM, p < 0.0001), and male patients exhibited significantly higher average ACE2 expression compared to female patients (6.0 vs 4.8 TPM, p < 0.0001). Pathway analysis shows that ACE2 is the most enriched gene in the renin-angiotensin pathway in male gender and underexpressed in patients older than 55 years (Supplementary Figs. 1, 2). Age- and gender-related ACE2 expression varied by cancer type, with significantly increased ACE2 expression in patients under 65 years for colorectal, gastric, and ovarian surface epithelial tumors, compared to patients 65 years and older (Supplementary Table 2). Despite the overall increased ACE2 expression in males, average male ACE2 expression was significantly increased in only NSCLC and salivary gland tumors, while ACE2 expression was significantly increased in female patients for many tumor types (Supplementary Table 3).

### ACE2 expression across tumor types

**Figure 1:**
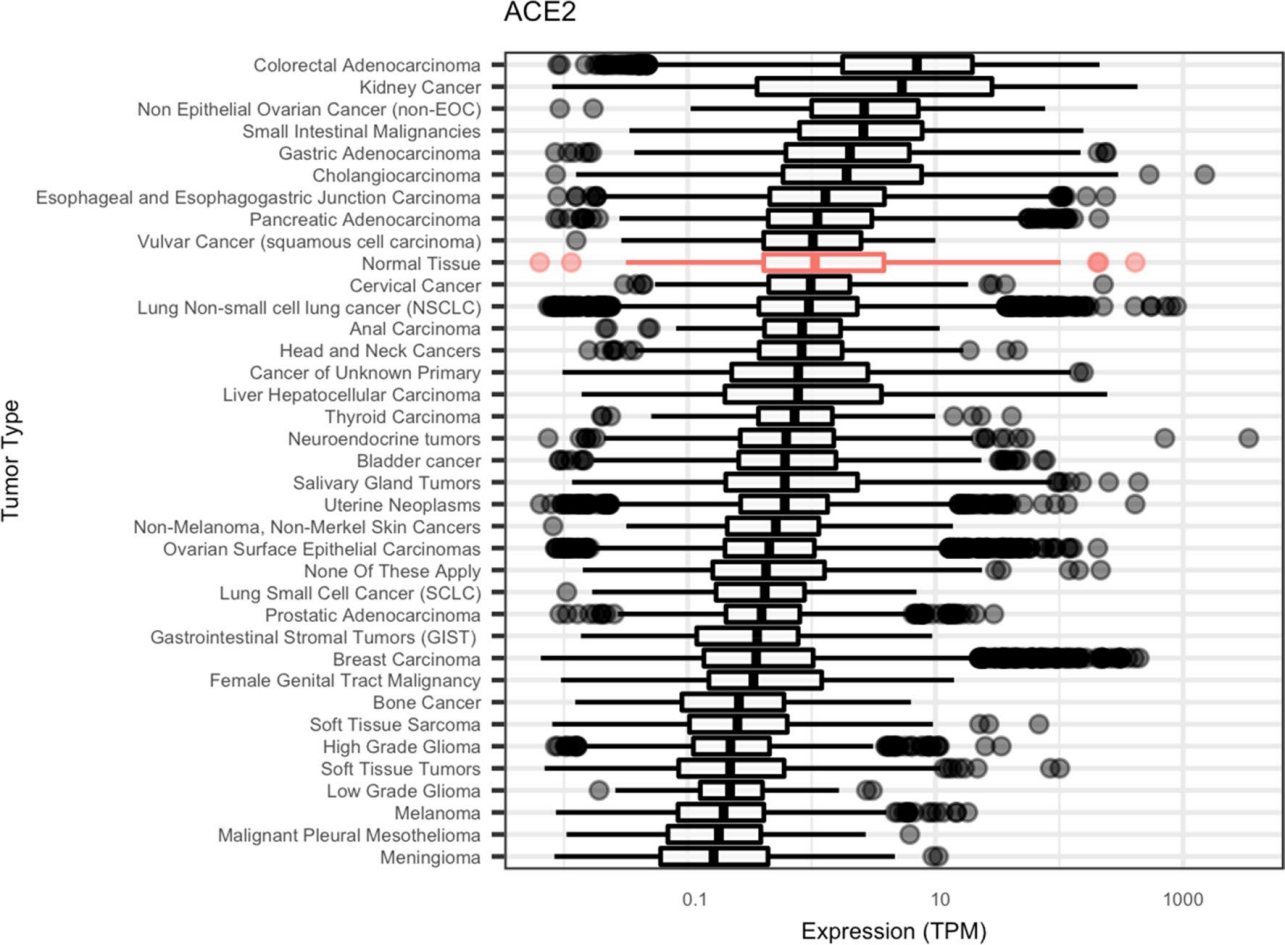
ACE2 gene expression in normal and cancerous tissues. Box plots indicate the median and upper/lower quartiles, with individual data points representing outlier values greater than 1.5 × the interquartile range above or below the upper or lower quartile, respectively.

Among the 36 tumor types investigated, median ACE2 expression was increased in nine tumor types compared to normal tissue (0.99 TPM), with the highest ACE2 expression observed in colorectal (7.08 TPM) and kidney (5.35 TPM) cancers (Fig. 1).

In addition to colorectal cancer, ACE2 expression in other gastrointestinal (GI) cancers, including small intestinal (2.62 TPM), gastric (2.04 TPM), cholangiocarcinoma (1.92 TPM), esophageal/esophagogastric junction (1.29 TPM), and pancreatic (1.11 TPM) cancers, was elevated relative to the composite normal tissue. Differences in tumor ACE2 expression compared the composite normal tissue were similar to those observed for tissue type-matched normal controls (Supplemental Fig. 3). While non-small cell lung cancer (NSCLC) exhibited slightly decreased median ACE2 expression (0.95 TPM) compared to normal tissue, a subset of NSCLC tumors (N = 64, 0.85%) demonstrated severely increased expression (> 50 TPM). The lowest ACE2 expression was observed in melanoma (0.19 TPM), mesothelioma (0.18 TPM), and meningioma (0.16 TPM). A similar pattern of ACE2 expression was seen across age- and gender-controlled subgroups (Supplemental Fig. 4), potentially suggesting that differences in ACE2 expression are largely attributed to tissue or cell type-specific expression.

### Protease gene expression across tumor types

**Figure 2:**
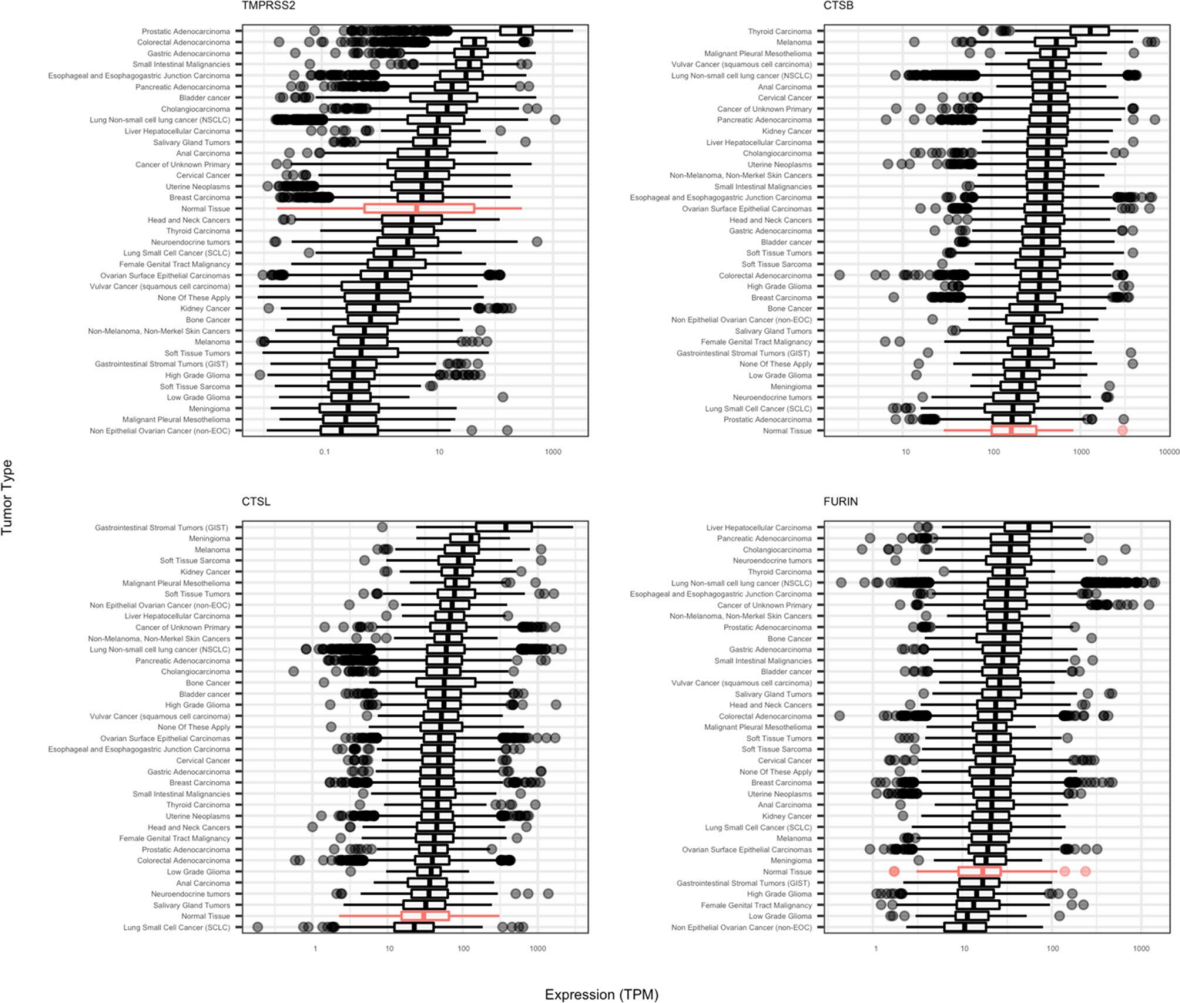
Protease gene expression in normal and cancerous tissues. Box plots indicate the median and upper/lower quartiles, with individual data points representing outlier values greater than 1.5 × the interquartile range above or below the upper or lower quartile, respectively.

Entry of SARS-CoV-2 into human cells is facilitated by priming of the viral spike protein by cellular proteases (Hoffman et al., 2020). While inhibition of the serine protease TMPRSS2 blocks SARS-CoV-2 infection of lung cells (Hoffman et al., 2020), other proteases, including cathepsin B and L (CTSB/CTSL) and FURIN, mediate spike protein priming and have cumulative effects with TMPRSS2 on viral entry (Shang et al. 2020). Median TMPRSS2 expression was increased in 16 tumor types, including NSCLC (10.35 TPM), compared to normal tissue (3.82 TPM), most notably in prostate cancer (265.43 TPM) (Fig. 2). Median tumor protease expression was similarly increased or decreased relative to tumor type-matched normal controls, with some exception where tumor expression was not significantly different from normal (Supplemental Figs. 5–8).

Similar to ACE2 expression, higher levels of TMPRSS2 expression was observed in many GI cancers (colorectal: 44.02 TPM, gastric: 40.12 TPM, small intestinal: 35.69 TPM, esophageal/esophagogastric junction: 31.16 TPM, pancreatic: 17.96 TPM, cholangiocarcinoma: 14.75 TPM, liver: 9.55 TPM, and anal: 6.78 TPM). CTSB, CTSL, and FURIN expression was increased in most tumor types relative to normal tissue. CTSB expression was highest in thyroid cancer (1287.97 TPM; normal: 166.61 TPM), CTSL expression was highest in gastrointestinal stromal tumors (376.26 TPM; normal: 30.36 TPM), and FURIN expression was highest in liver cancer (55.37 TPM; normal: 16.89 TPM). Consistent with ACE2 expression, expression levels for each protease showed a similar pattern across tumor types in age- and gender-controlled subgroups (Supplemental Figs. 9–12).

### Co-expression of ACE2 and key protease genes across tumor types

**Figure 3:**
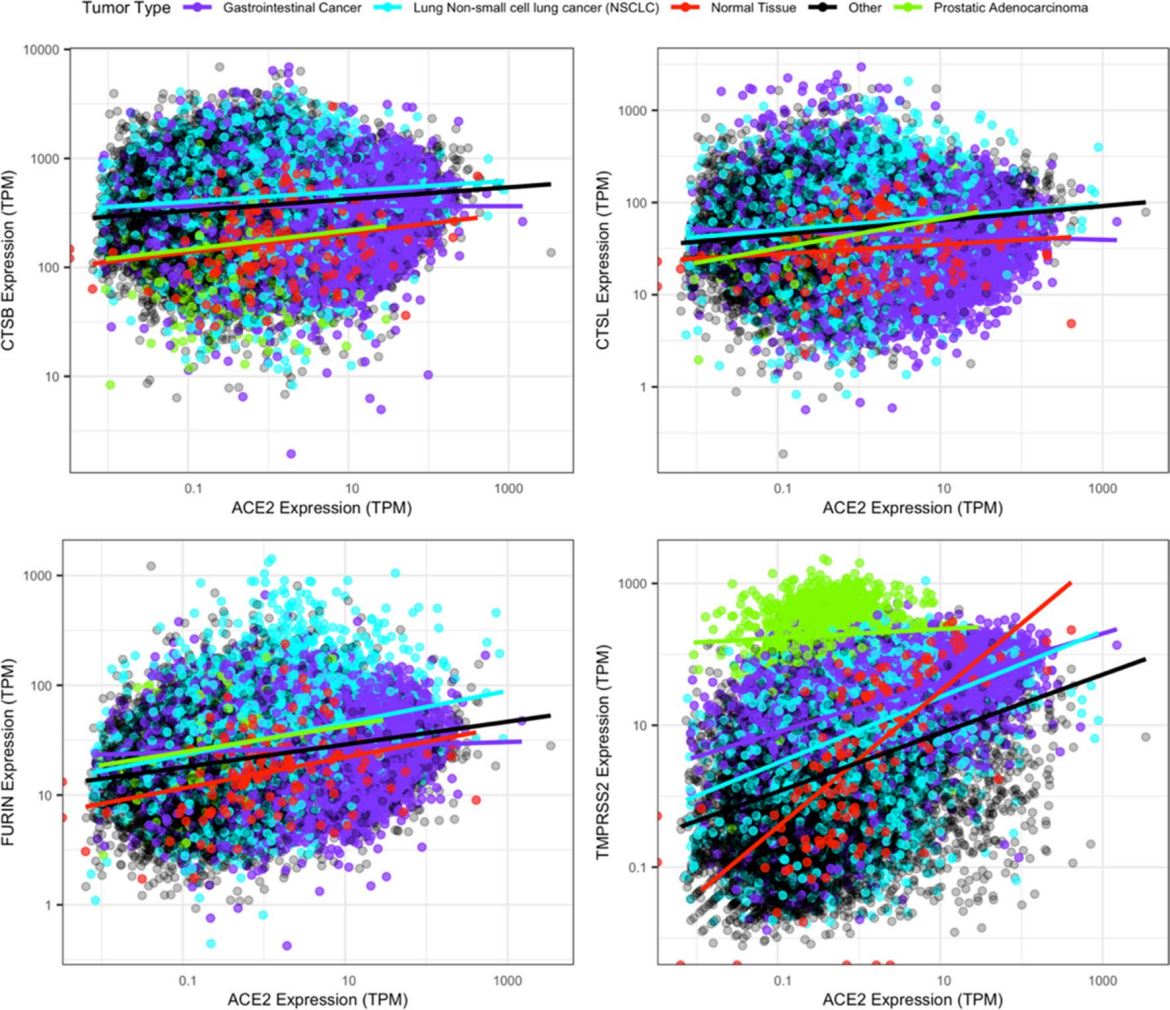
Co-expression of ACE2 and protease genes in normal and cancerous tissues. Data points are color-coded for normal (red), gastrointestinal cancers (purple), NSCLC (cyan), prostate cancer (green), and all other cancer types (black). Regression lines represent log-transformed fits.

We examined the co-expression of ACE2 with key protease genes to evaluate the correlation between ACE2 and protease gene expression (Fig. 3).

In normal tissue, the correlation with ACE2 expression was strongest for TMPRSS2 (Pearson’s correlation [r] = 0.44, slope [m] = 0.95) compared to CSTB (r = 0.09, m = 0.09), CSTL (r = − 0.10, m = 0.05), and FURIN (r = − 0.04, m = 0.14). Interestingly, most tumor types exhibited weaker ACE2/TMPRSS2 and ACE2/CTSB correlations compared to normal tissue, whereas the ACE2/CTSL and ACE2/FURIN correlations in normal tissue are weaker than that of most cancer types. Tumor type-specific deviations from the primary ACE2/TMPRSS2 cluster were observed for prostate (r = − 0.05, m = 0.06) and GI cancers (r = 0.27, m = 0.35), while expression in NSCLC tumors (r = 0.07, m = 0.46) largely overlapped with the primary cluster. Tumor type-specific deviations in ACE2/protease co-expression were less prominent for CTSB, CTSL, and FURIN compared to TMPRSS. Notably, ACE2/protease co-expression in GI cancers deviated from the primary cluster due to increased ACE2 expression (CTSB: r = − 0.02, m = − 0.0001; CTSL: r = − 0.04, m = − 0.02; FURIN: r = 0.03, m = 0.02). Together, this suggests the co-expression of ACE2 and protease genes may be more predictive of susceptibility to viral infection by SARS-CoV-2 than ACE2 expression alone, with GI cancers exhibiting the highest potential susceptibility.

### ACE2 expression and association with cell population abundance in the TME in NSCLC

**Figure 4:**
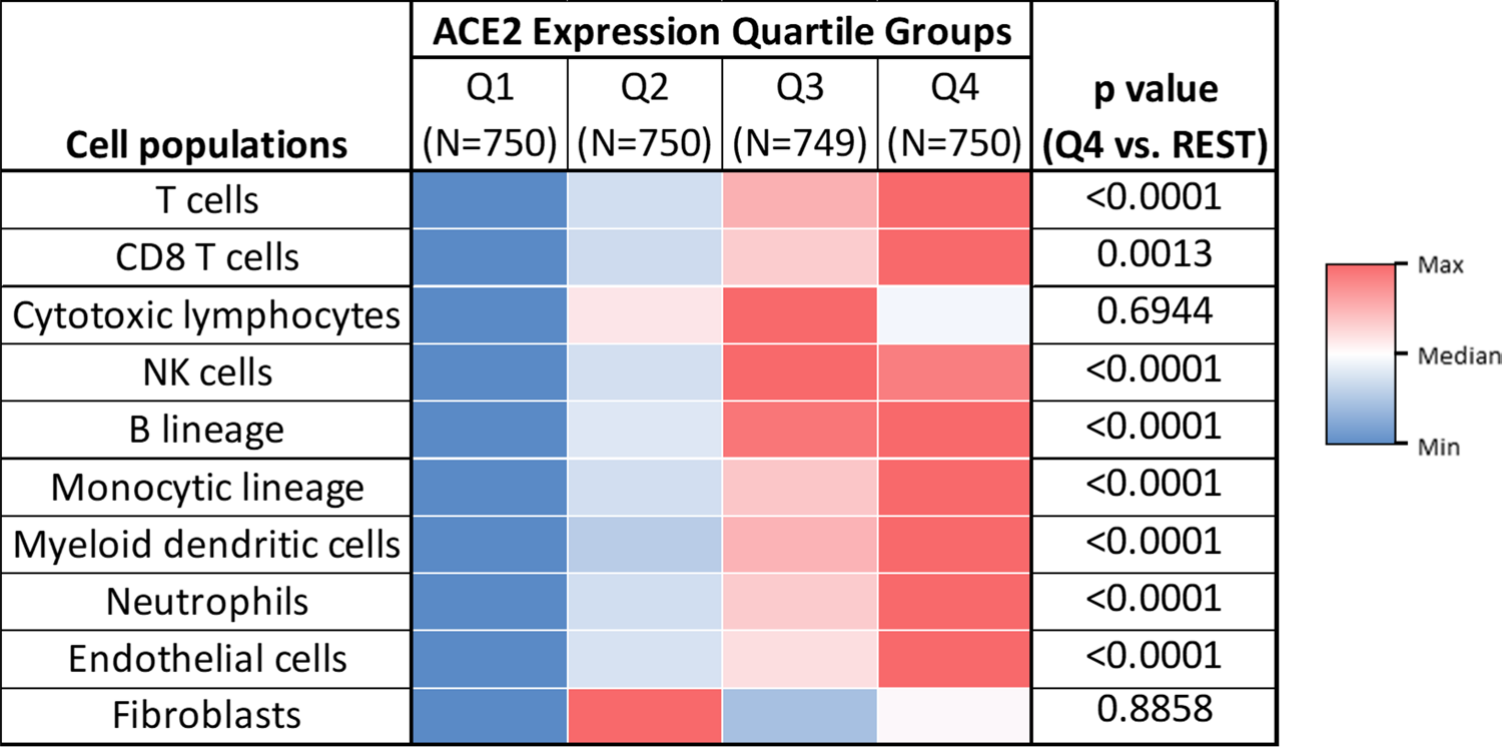
Association between ACE2 expression and estimated immune cell infiltration in NSCLC tumors. MCP-counter results for immune and stromal cell population abundance in subgroups based on ACE2 expression. Heatmap indicates relative cell population abundance, with values color-coded by maximum (red), minimum (blue), and median (white).

In addition to its function as a receptor for SARS-CoV-2, ACE2 is suggested to be involved in various aspects of post-infection processes, and the expression of ACE2 in lung has been shown to associate with innate and acquired immune responses^26^. We used the Microenvironment Cell Populations (MCP)-counter^27^ to estimate cell population abundance in the TME, inferred by RNA expression tested by WTS in NSCLC tumors of adenocarcinoma histology. Consistent with the previous observation of ACE2 expression level correlating with neutrophils, NK cells and various Th cells in SARS-CoV infected cells^26^, we observed in NSCLC tumor tissues significant positive correlations of ACE2 expression with T cells, NK cells, B cells, monocytic lineage, dendritic cells, neutrophils, as well as endothelial cells (p < 0.0001), Fig. 4.

## Discussion

The study presented here provides, to our knowledge, the first systematic assessment of RNA expression of key molecules involved in the infectious process leading to COVID-19 in advanced cancer using data from a large cohort of patients who have undergone routine molecular profiling of their malignancies. We found extensive variability of expression of the main receptor for SARS-CoV-2, ACE2, in particular in NSCLC and gastrointestinal cancers. Our findings are of immediate relevance for an improved understanding of the dynamics of the current COVID-19 pandemic since recent studies highlight the particular vulnerability of patients with cancer in terms of increased mortality but also worse severity of the disease^3,4^. While there are manifold potential pathophysiologic underpinnings for this concerning finding, including immune-suppression and smoking history, differential expression of the primary viral receptor in normal and malignant tissues might contribute. Knowledge of such systematic differences could lead to improved risk prediction for patients with cancer.

Our analysis of normal tissue samples demonstrates significantly higher ACE2 levels in males and in patients of age less than 65 compared to females and older patients, respectively. This finding mirrors the clinical observation of increased mortality and morbidity in male patients in Asian and Western populations while not in agreement with the worse outcomes from the infection in older patients^2,28,29^. Thus, it is conceivable that differences in expression levels of ACE2 in normal tissues contribute to differences in risk of infection and disease severity in men but also highlight the possible impact of co-morbidities in the elderly. Interestingly, multiple components of the renin-angiotensin system appear to be co-regulated with ACE2 in normal tissues, indicating that there is a potential complex interplay between the infection and pre-existing conditions, which might contribute to the worse outcome for patient with hypertension and other cardiovascular diseases^28^. ACE2 expression is also regulated post-transcriptionally, including down-regulation by miR-421 via disruption of ACE2 translation^30^, and post-translationally, with shedding of ACE2 from cell membranes regulated by metalloproteinase ADAM17^31^. Thus, transcriptional expression of ACE2 may not be concordant with ACE2 protein level in some instances.

Examination of our extensive database of molecular profiles obtained from patients with advanced malignancies revealed wide-ranging variability in expression of ACE2 in samples from advanced cancers. Highest ACE2 RNA expression levels were observed in colorectal, renal, and NSCLC cancers as well as in cholangio- and gastric carcinomas. These findings are in agreement with previous observations in the TCGA dataset, which consists mostly of data from resected, non-metastatic tumors^17^ while our dataset is overwhelmingly derived from patients with advanced cancers. Our analysis of expression levels of several proteases known to function as coreceptors demonstrates variable correlation with ACE2. Correlation of ACE2 expression with protease expression was particularly pronounced in GI cancers suggesting these patients may have heightened susceptibility for SARS-CoV-2 infection and a more severe course of COVID-19. From a clinical perspective, of particular interest is our finding that subgroups of patients with intensely increased ACE2 expression levels exist in several cancer types, including NSCLC and breast cancers, as well as in glioblastoma and melanoma. While, in general, little is known about the interaction of human viruses with malignant tumors, data from studies of oncolytic viruses demonstrate that viruses can replicate in malignant masses and sustain ongoing viremia if viral receptors are present on tumor cells^32^. Thus, it is conceivable that malignant tumors with particularly high ACE2 and protease expression function as viral reservoirs leading to sustained viremia and increased disease severity. This hypothesis needs to be tested clinically since it would suggest intensified isolation measures or even prophylactic antiviral treatment for patients with tumors that express ACE2 at very high level. In addition, our finding of increased presence of various immune-cell lineages, including T cells and NK cells, in tumors with high ACE2 expression highlights the possibility that such tumors are not only susceptible to SARS-CoV-2 infection but could also promote the severe inflammatory response observed in many patients with COVID-19. On the other hand, intratumoral injection of flu vaccine can promote conversion of “cold” tumors to “hot”, generates systemic CD8 + T cell-mediated antitumor immunity, and sensitizes resistant tumors to checkpoint blockade^33^. Thus, one could speculate that, in some cases, SARS-CoV-2 infection might promote tumor regression in the context of immune checkpoint inhibitors.

In summary, our investigation demonstrates significant differences in ACE2 and protease expression in normal and malignant tissues with a subgroup of patients expressing very high levels of ACE2 RNA. These findings, together with the increased presence of inflammatory cells in tumors displaying high ACE2 levels might contribute to the developing complex pathophysiologic picture of COVID-19 and help guide prophylactic measures in patients with solid malignancies.

## Supplementary information

Supplementary information.
