# Supplementary material for: Pan-cancer analysis of RNA expression of ANGIOTENSIN-I-CONVERTING ENZYME 2 reveals high variability and possible impact on COVID-19 clinical outcomes
Source: Sci Rep. 2021 Mar 11;11:5639. doi: 10.1038/s41598-021-84731-7 (PMC7952699; doi:10.1038/s41598-021-84731-7)
Supplement: Supplementary file 1 — Supplementary information. [file 41598_2021_84731_MOESM1_ESM.pdf]

Pan-cancer analysis of RNA expression of ANGIOTENSIN-I-CONVERTING ENZYME 2 reveals high variability and possible impact on COVID-19 clinical outcomes

Andrew Elliott[1], Michelle Saul[1], Jia Zeng[1], John L. Marshall[2], Edward S. Kim[3], Misako Nagasaka[4], Heinz-Josef Lenz[5], Lee Schwartzberg[6], David Spetzler[1], Jim Abraham[1], Joanne Xiu[1], Phillip Stafford[1], and W. Michael Korn[1,7,8]

1. Caris Life Sciences, Phoenix, AZ, USA
2. Ruesch Center for The Cure of Gastrointestinal Cancers, Lombardi Comprehensive Cancer Center, Georgetown University Medical Center, Washington, DC, USA
3. Levine Cancer Institute, Atrium Health, Charlotte, NC, USA
4. Department of Oncology, Karmanos Cancer Institute, Wayne State University, Detroit, MI, USA
5. University of Southern California, Keck School of Medicine, Norris Comprehensive Cancer Center, Los Angeles, California
6. Medical Oncology, West Cancer Center, 9745 Wolf River Blvd, Germantown, TN, USA
7. Division of Hematology/Oncology, Department of Medicine, University of California San Francisco, San Francisco, CA, USA

8. Corresponding Author:

W. Michael Korn, MD

4610 South 44th Place

Phoenix, AZ 85040

Phone: (602) 464-7537

Email addresses: wmkorn@carisls.com

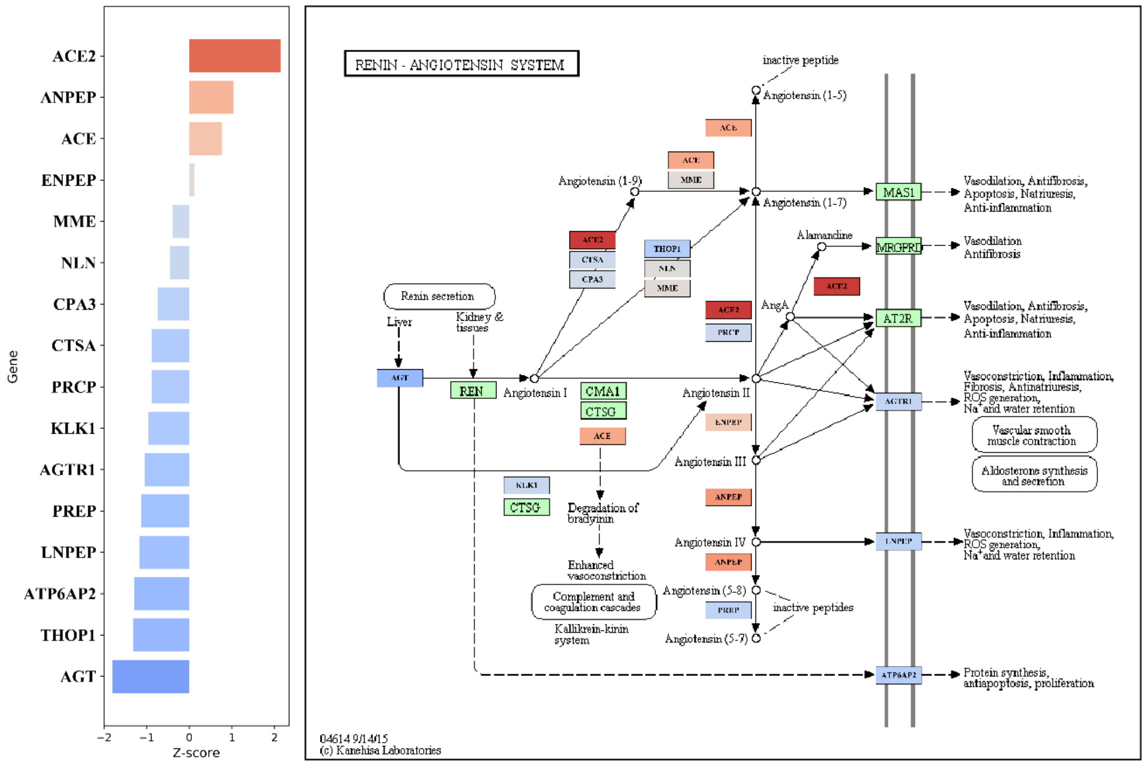

Supplementary Figure 1: KEGG color mapping of Renin-angiotension pathway in male vs. female in non-tumor tissues. Z-scores reflect the differential gene expression between groups relative to the average differential expression, with red and blue colors indicating increased expression in male and female groups, respectively. Among all the genes in the pathway, the fold change of ACE2 expression in the male group compared to the female group is the highest.

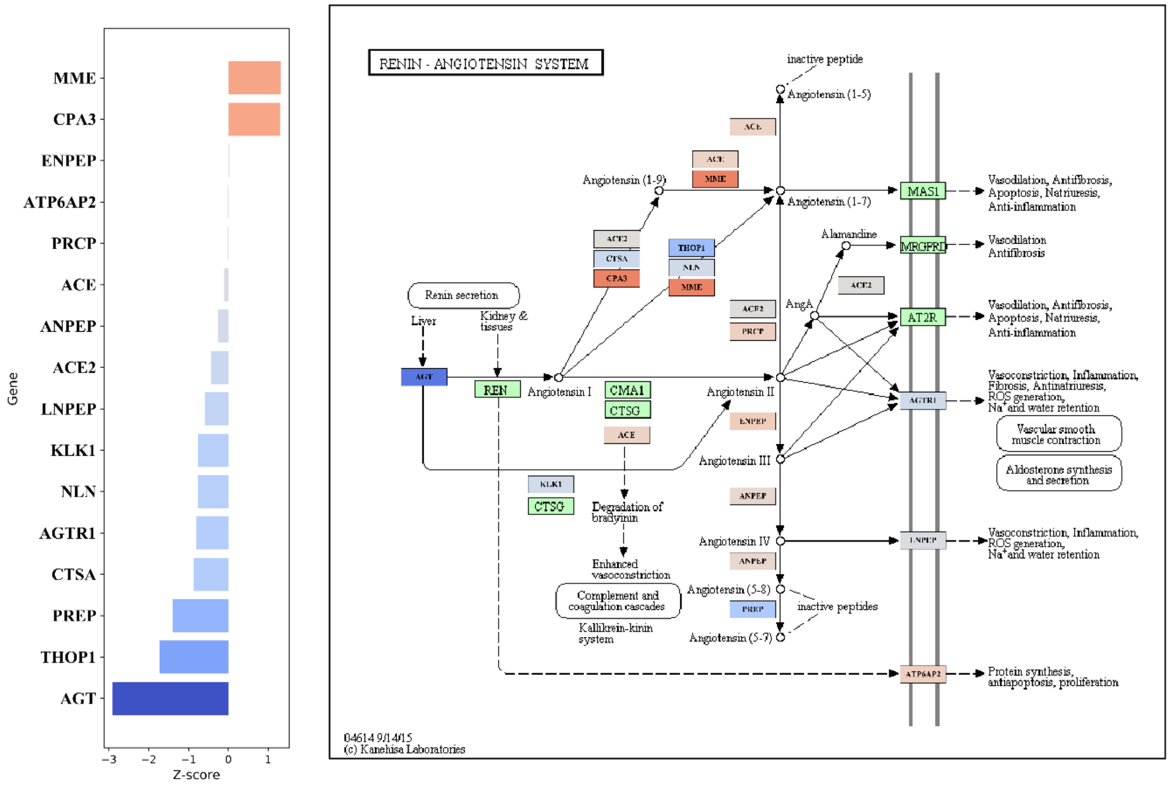

Supplementary Figure 2: KEGG color mapping of Renin-angiotension pathway in old vs. young patients in non-tumor tissues. Z-scores reflect the differential gene expression between groups relative to the average differential expression, with red and blue colors indicating increased expression in >= 55 and < 55 years old groups, respectively. ACE2 is under-expressed in the group of age >= 55 years old at tissue collection, compared to the group of age < 55 years old.

Cancer Type	N samples	% of Total
Bladder cancer	4	3.1%
Breast Carcinoma	14	11.0%
Cancer of Unknown Primary	1	0.8%
Cervical Cancer	2	1.6%
Cholangiocarcinoma	2	1.6%
Colorectal Adenocarcinoma	35	27.6%
Gastric Adenocarcinoma	1	0.8%
Head and Neck Cancers	3	2.4%
High Grade Glioma	2	1.6%
Lung Non-small cell lung cancer (NSCLC)	8	6.3%
Male Genital Tract Malignancy	1	0.8%
Melanoma	6	4.7%
Merkel Cell Carcinoma (MCC)	1	0.8%
Non-Melanoma, Non-Merkel Skin Cancers	2	1.6%
Ovarian Surface Epithelial Carcinomas	9	7.1%
Pancreatic Adenocarcinoma	17	13.4%
Pituitary carcinomas, Oligodendroglioma	1	0.8%
Prostatic Adenocarcinoma	1	0.8%
Salivary Gland Tumors	2	1.6%
Small Intestinal Malignancies	1	0.8%
Soft Tissue Tumors	2	1.6%
Thyroid Carcinoma	1	0.8%
Uterine Neoplasms	11	8.7%
**Total**	**127**	**100.0%**

Supplementary Table 1: Distribution of tumor types for molecularly-profiled adjacent normal tissue.

Supplemental Table 2								
**Tumor Type**	**Age group**	**N samples**	**Mean**	**≥65/<65 Mean**	**Median**	**Range**	**p-value**	**FDR p-value**
Anal Carcinoma	≥65	58	1.401	0.93525	0.8388125	0.116429-10.3971	0.9442	0.986429
	<65	88	1.498		0.838141	0.021452-10.8124		
Bladder cancer	≥65	911	1.865	0.98992	0.604232	0.009124-77.2456	0.34265	0.705674
	<65	340	1.884		0.6199005	0.017229-36.9566		
Bone Cancer	≥65	18	0.8784	1.51266	0.206812	0.017246-6.34352	0.61989	0.889915
	<65	66	0.5807		0.2875205	0.012325-4.80362		
Breast Carcinoma	≥65	1300	3.907	1.236	0.3547435	0.007306-441.295	0.84009	0.939381
	<65	2408	3.161		0.358131	0.006494-356.919		
Cancer of Unknown Primary	≥65	526	4.306	0.94846	0.7886195	0.009711-156.715	0.66571	0.889915
	<65	455	4.54		0.773087	0.014241-124.121		
Cervical Cancer	≥65	96	1.882	0.80773	0.9817895	0.038238-16.5094	0.99606	0.996534
	<65	490	2.33		0.982306	0.030627-227.08		
Cholangiocarcinoma	≥65	568	12.48	1.25756	1.977715	0.012502-1495.36	0.11301	0.43526
	<65	489	9.924		1.77278	0.008565-532.834		
Colorectal Adenocarcinoma	≥65	2200	14.41	0.90915	6.234825	0.009376-206.638	3E-05	0.001034
	<65	3462	15.85		7.69504	0.008912-211.35	****	**
Esophageal and Esophagogastric Junction Carcinoma	≥65	556	5.458	0.85495	1.23281	0.012509-113.032	0.15002	0.470883
	<65	509	6.384		1.38746	0.008903-237.214		
Female Genital Tract Malignancy	≥65	112	0.9577	0.82489	0.3142375	0.009449-14.1333	0.30625	0.677877
	<65	85	1.161		0.394063	0.010617-11.137		
Gastric Adenocarcinoma	≥65	334	6.08	0.65701	1.54679	0.008494-235.156	0.00291	0.023882
	<65	388	9.254		2.547735	0.010704-240.038	**	*
Gastrointestinal Stromal Tumors (GIST)	≥65	59	0.7672	0.94262	0.370421	0.02015-4.90965	0.90647	0.96141
	<65	89	0.8139		0.351213	0.013688-9.40761		
Head and Neck Cancers	≥65	264	1.418	0.8537	0.8484825	0.026259-16.7958	0.69047	0.889915
	<65	351	1.661		0.799541	0.015797-46.0084		
High Grade Glioma	≥65	320	0.5665	1.11802	0.2194545	0.011017-25.2778	0.38962	0.714315
	<65	913	0.5067		0.221776	0.008511-33.9888		
Kidney Cancer	≥65	157	25.19	1.06467	10.8534	0.011104-215.118	0.03149	0.18371
	<65	276	23.66		3.582875	0.008018-428.784	*	
Liver Hepatocellular Carcinoma	≥65	93	11.21	1.28452	0.841662	0.037577-243.748	0.44204	0.748613
	<65	94	8.727		0.760869	0.013854-204.511		
Low Grade Glioma	≥65	13	0.3336	0.88629	0.29198	0.036145-0.95256	0.64217	0.889915
	<65	109	0.3764		0.209425	0.019205-3.096		
Lung Non-small cell lung cancer (NSCLC)	≥65	4676	3.41	0.91006	0.975731	0.007438-720.056	0.59562	0.889915
	<65	2854	3.747		0.906848	0.007748-883.672		

Supplemental Table 2 (cont.)

Tumor Type	Age group	N samples	Mean	≥65/<65 Mean	Median	Range	p-value	FDR p-value
Lung Small Cell Cancer (SCLC)	≥65	166	0.7842	1.03211	0.414097	0.023891-7.02712	0.78111	0.898968
	<65	161	0.7598		0.418892	0.010532-6.97318		
Malignant Pleural Mesothelioma	≥65	64	0.3815	1.5949	0.179445	0.010902-6.20635	0.99653	0.996534
	<65	27	0.2392		0.178203	0.010483-0.83776		
Melanoma	≥65	418	0.6295	1.41747	0.1912205	0.0087-18.2022	0.66646	0.889915
	<65	347	0.4441		0.201832	0.008599-8.74036		
Meningioma	≥65	29	0.6381	0.83227	0.231594	0.015256-4.70228	0.56442	0.889915
	<65	52	0.7667		0.161024	0.008361-10.5617		
Neuroendocrine tumors	≥65	216	1.434	0.08901	0.639484	0.007446-25.1163	0.85103	0.940616
	<65	293	16.11		0.59655	0.015035-3385.94		
Non Epithelial Ovarian Cancer (non-EOC)	≥65	14	8.832	2.14577	3.19119	0.105422-76.8812	0.84097	0.939381
	<65	69	4.116		2.58899	0.009258-18.2563		
None Of These Apply	≥65	59	5.271	1.35746	0.397129	0.032733-215.854	0.30418	0.677877
	<65	122	3.883		0.4697485	0.014173-143.732		
Non-Melanoma, Non-Merkel Skin Cancers	≥65	85	1.189	1.54315	0.537467	0.008175-13.8366	0.33158	0.705674
	<65	51	0.7705		0.480838	0.037419-3.86034		
Ovarian Surface Epithelial Carcinomas	≥65	1708	1.026	0.45865	0.389794	0.008823-128.479	1E-16 *****	1.05E-14 *****
	<65	1935	2.237		0.53348	0.00849-204.636		
Pancreatic Adenocarcinoma	≥65	1195	4.508	1.33928	1.14089	0.008563-208.825	0.28477	0.677877
	<65	909	3.366		1.08737	0.013642-112.933		
Prostatic Adenocarcinoma	≥65	642	0.9158	1.03038	0.395661	0.009242-21.2936	0.96134	0.989613
	<65	465	0.8888		0.39438	0.019949-29.5996		
Salivary Gland Tumors	≥65	94	15.27	3.2517	0.6415905	0.016091-435.871	0.73787	0.889915
	<65	118	4.696		0.585181	0.011613-98.5849		
Small Intestinal Malignancies	≥65	137	8.567	1.21604	2.6824	0.033752-156.718	0.74583	0.889915
	<65	159	7.045		2.57623	0.037853-60.3695		
Soft Tissue Sarcoma	≥65	48	2.585	2.53929	0.289598	0.010082-68.5051	0.34276	0.705674
	<65	56	1.018		0.21899	0.008025-27.0002		
Soft Tissue Tumors	≥65	202	0.6777	0.45361	0.193441	0.007722-15.3721	0.15248	0.470883
	<65	343	1.494		0.236406	0.006963-101.023		
Thyroid Carcinoma	≥65	102	1.442	0.79933	0.643828	0.020029-19.9003	0.36363	0.714315
	<65	119	1.804		0.828351	0.050606-41.2267		
Uterine Neoplasms	≥65	1203	1.69	1	0.655819	0.00789-116.546	0.09334	0.408383
	<65	1195	1.69		0.57353	0.006422-409.876		
Vulvar Cancer (squamous cell carcinoma)	≥65	41	2.052	1.31707	1.09109	0.012548-10.0039	0.30989	0.677877
	<65	57	1.558		1.01655	0.028997-7.82711		

Supplemental Table 2: ACE2 expression in patients ≥ 65 years and < 65 years by tumor type. *P<0.05, **P<0.01, ***P<0.001, ****P<0.0001*****P<0.00001 (Wilcoxon test). FDR p-values are adjusted for multiple comparisons (Benjamini-Hochberg).

Supplemental Table 3

Tumor Type	Gender	N samples	Mean	Male/ Female Mean	Median	Range	p-value	FDR p-value
Anal Carcinoma	Male	38	1.57	1.10486	0.8492615	0.022517-10.8124	0.90592	0.96141
	Female	108	1.421		0.8314885	0.021452-10.3971		
Bladder cancer	Male	875	1.68	0.72696	0.546908	0.009304-73.579	0.00056 ***	0.010115 *
	Female	376	2.311		0.7494495	0.009124-77.2456		
Bone Cancer	Male	38	0.7557	1.36754	0.2417185	0.012948-6.34352	0.63876	0.889915
	Female	46	0.5526		0.2692035	0.012325-3.24048		
Breast Carcinoma	Male	29	0.4321	0.12539	0.151944	0.017469-5.93741	0.00092 ***	0.010745 *
	Female	3679	3.446		0.359318	0.006494-441.295		
Cancer of Unknown Primary	Male	482	4.687	1.12913	0.727244	0.010493-144.231	0.48958	0.803219
	Female	499	4.151		0.851373	0.009711-156.715		
Cervical Cancer	Male	0	NA	NA	NA	NA	NA	NA
	Female	586	2.257		0.982306	0.030627-227.08		
Cholangiocarcinoma	Male	481	8.79	0.65646	1.84517	0.008565-298.722	0.2342	0.630538
	Female	576	13.39		1.974205	0.012502-1495.36		
Colorectal Adenocarcinoma	Male	3139	14.74	0.9224	7.01268	0.008912-190.094	0.29476	0.677877
	Female	2523	15.98		7.15583	0.009376-211.35		
Esophageal and Esophagogastric Junction Carcinoma	Male	875	5.74	0.86446	1.25415	0.008903-237.214	0.05868	0.308067
	Female	190	6.64		1.546115	0.036392-113.032		
Female Genital Tract Malignancy	Male	0	NA	NA	NA	NA	NA	NA
	Female	197	1.045		0.338948	0.009449-14.1333		
Gastric Adenocarcinoma	Male	429	6.147	0.60383	1.49358	0.010704-148.469	0.00044 ***	0.010115 *
	Female	293	10.18		2.72578	0.008494-240.038		
Gastrointestinal Stromal Tumors (GIST)	Male	88	0.7654	0.91217	0.307182	0.02015-9.40761	0.0785	0.35838
	Female	60	0.8391		0.4876625	0.013688-4.90965		
Head and Neck Cancers	Male	475	1.392	0.65784	0.746642	0.015797-46.0084	0.00067 ***	0.010115 *
	Female	140	2.116		1.136375	0.024457-37.4005		
High Grade Glioma	Male	761	0.4575	0.73025	0.206038	0.008511-25.2778	0.00077 ***	0.010116 *
	Female	472	0.6265		0.2645565	0.012672-33.9888		
Kidney Cancer	Male	317	22.15	0.7413	5.35429	0.008018-428.784	0.77841	0.898968
	Female	116	29.88		5.3639	0.011325-245.99		
Liver Hepatocellular Carcinoma	Male	148	10.31	1.19301	0.7729625	0.018889-243.748	0.77871	0.898968
	Female	39	8.642		0.809191	0.013854-85.8406		
Low Grade Glioma	Male	62	0.3901	1.1051	0.230156	0.019205-3.096	0.35797	0.714315
	Female	60	0.353		0.210395	0.025964-1.63778		
Lung Non-small cell lung cancer (NSCLC)	Male	3760	3.818	1.17188	0.840921	0.007438-883.672	3.6E-13 *****	1.89E-11 *****
	Female	3770	3.258		1.078485	0.008252-555.852		

Supplemental Table 3 (cont.)								
**Tumor Type**	**Gender**	**N samples**	**Mean**	**Male/ Female Mean**	**Median**	**Range**	**p-value**	**FDR p-value**
Lung Small Cell Cancer (SCLC)	Male	171	0.7856	1.03696	0.413373	0.010532-6.97318	0.70567	0.889915
	Female	156	0.7576		0.419363	0.023891-7.02712		
Malignant Pleural Mesothelioma	Male	58	0.2434	0.47942	0.1470985	0.010902-1.38278	0.12366	0.43526
	Female	33	0.5077		0.211113	0.010483-6.20635		
Melanoma	Male	482	0.4676	0.68988	0.1744365	0.008599-18.2022	0.00058 ***	0.010115 *
	Female	283	0.6778		0.237895	0.011874-14.838		
Meningioma	Male	29	1.038	1.9102	0.153868	0.016059-10.5617	0.69719	0.889915
	Female	52	0.5434		0.1695085	0.008361-4.70228		
Neuroendocrine tumors	Male	262	4.451	0.28441	0.5993215	0.007446-711.75	0.66659	0.889915
	Female	247	15.65		0.628837	0.015035-3385.94		
Non Epithelial Ovarian Cancer (non-EOC)	Male	0	NA	NA	NA	NA	NA	NA
	Female	83	4.912		2.65351	0.009258-76.8812		
None Of These Apply	Male	97	2.887	0.48053	0.409862	0.030333-119.811	0.50473	0.815328
	Female	84	6.008		0.4758545	0.014173-215.854		
Non-Melanoma, Non-Merkel Skin Cancers	Male	90	0.8861	0.67231	0.486375	0.031875-7.20834	0.37098	0.714315
	Female	46	1.318		0.746896	0.008175-13.8366		
Ovarian Surface Epithelial Carcinomas	Male	3	6.833	NA	1.57205	1.34999-17.5779	NA	NA
	Female	3640	1.665		0.454426	0.00849-204.636		
Pancreatic Adenocarcinoma	Male	1129	4.026	1.006	1.01971	0.016859-130.159	0.00266 **	0.023882 *
	Female	975	4.002		1.30434	0.008563-208.825		
Prostatic Adenocarcinoma	Male	1106	0.9049	NA	0.3945765	0.009242-29.5996	NA	NA
	Female	1	0.3718		0.371784	0.371784-0.371784		
Salivary Gland Tumors	Male	126	13.74	4.58153	0.7829865	0.011613-435.871	0.00296 **	0.023882 *
	Female	86	2.999		0.442164	0.016091-98.5849		
Small Intestinal Malignancies	Male	167	6.381	0.6702	2.16051	0.033752-68.8662	0.01884 *	0.126995
	Female	129	9.521		3.09258	0.039024-156.718		
Soft Tissue Sarcoma	Male	40	2.892	2.82975	0.264826	0.008025-68.5051	0.73077	0.889915
	Female	64	1.022		0.231236	0.013008-22.6927		
Soft Tissue Tumors	Male	283	0.9205	0.62028	0.202173	0.007722-84.5198	0.01138 *	0.085344
	Female	262	1.484		0.2557945	0.006963-101.023		
Thyroid Carcinoma	Male	93	1.315	0.70283	0.60673	0.020029-14.0587	0.10329	0.433834
	Female	128	1.871		0.820253	0.055852-41.2267		
Uterine Neoplasms	Male	2	1.007	NA	1.0072355	0.824561-1.18991	NA	NA
	Female	2396	1.691		0.6045	0.006422-409.876		
Vulvar Cancer (squamous cell carcinoma)	Male	0	NA	NA	NA	NA	NA	NA
	Female	98	1.765		1.023205	0.012548-10.0039		

Supplemental Table 3: ACE2 expression in male and female patients by tumor type. *P<0.05, **P<0.01, ***P<0.001, ****P<0.0001*****P<0.00001 (Wilcoxon test). FDR p-values are adjusted for multiple comparisons (Benjamini-Hochberg).

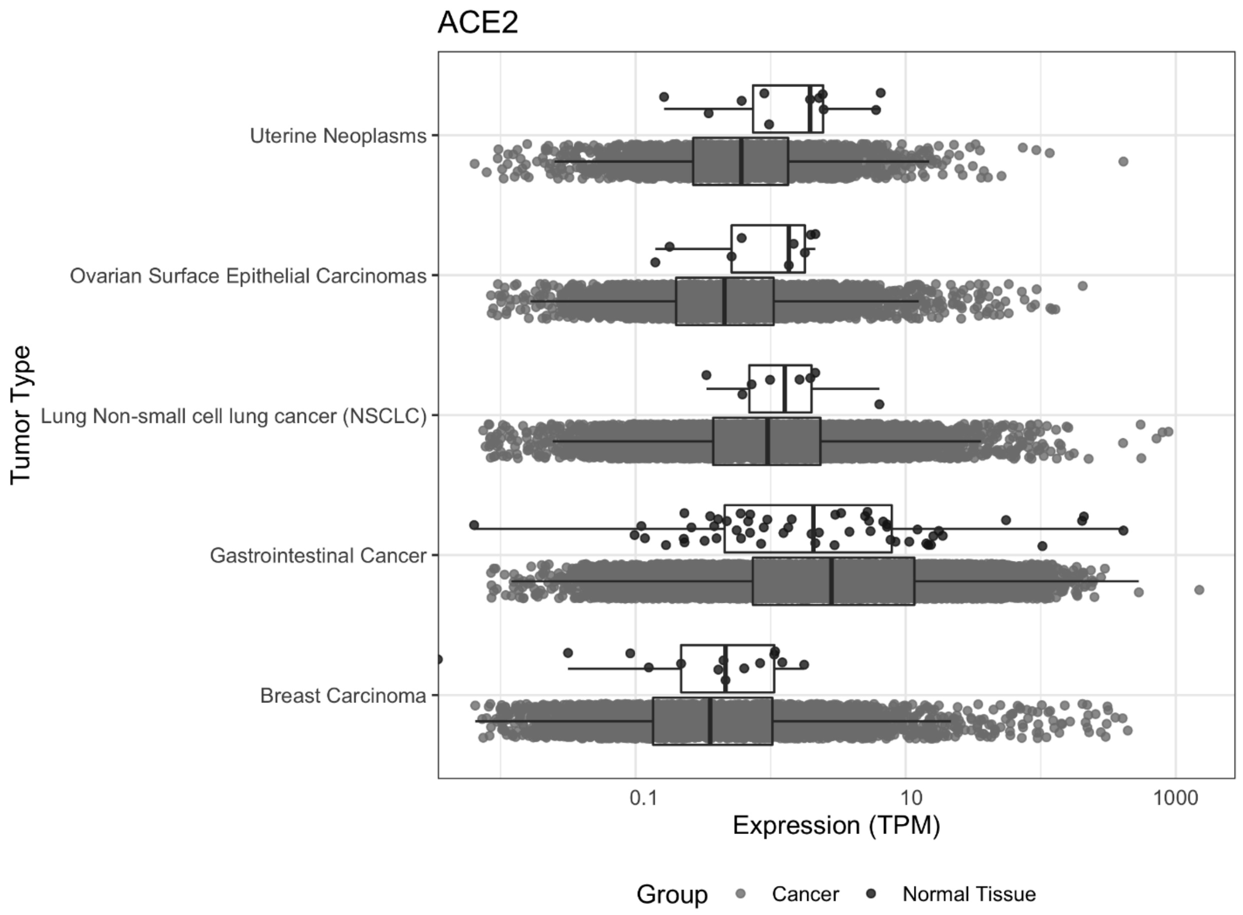

Supplemental Figure 3: ACE2 gene expression in tissue type-matched normal and cancerous tissues. Box plots indicate the median and upper/lower quartiles, with individual data points above/below the whiskers representing outlier values greater than 1.5x the interquartile range above or below the upper or lower quartile, respectively.

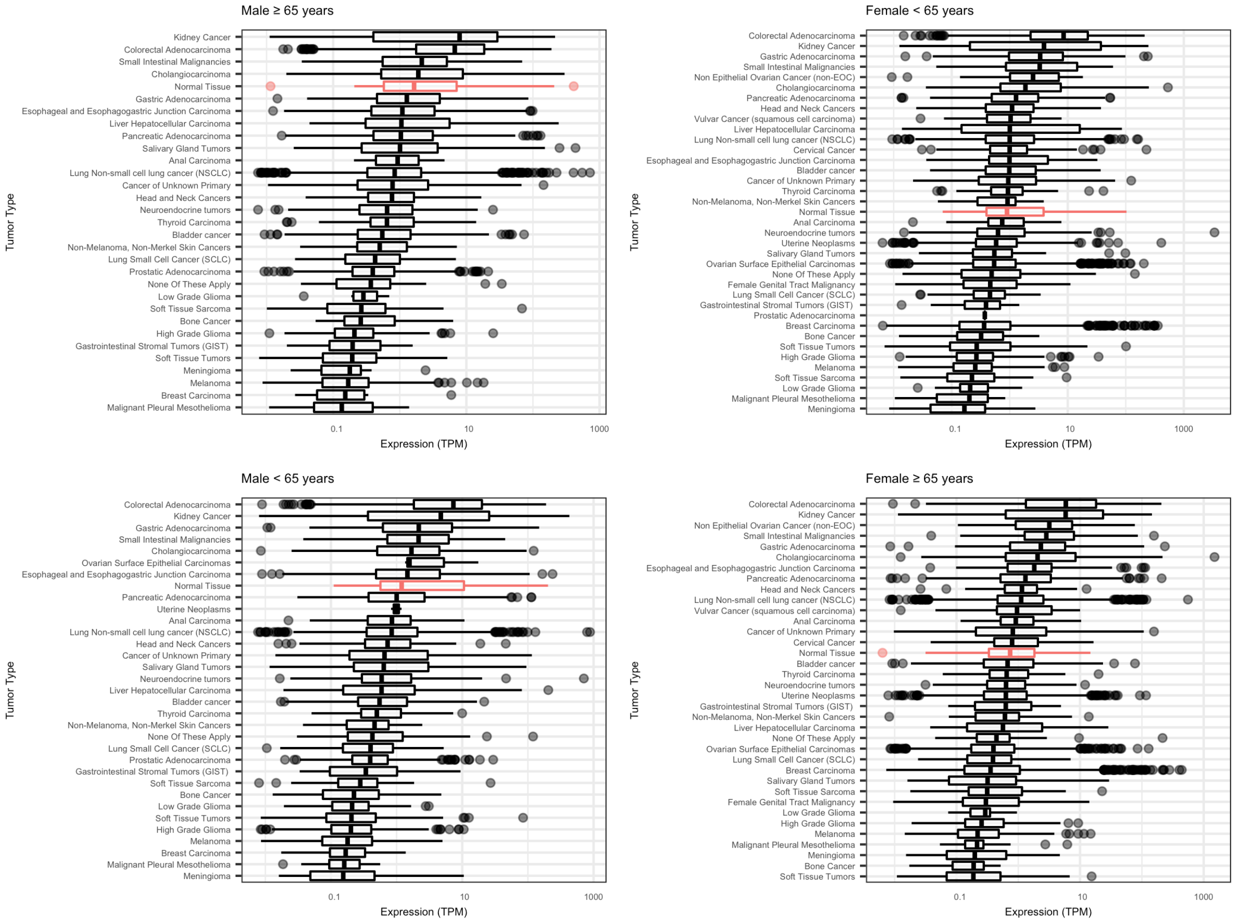

Supplementary Figure 4: ACE2 gene expression in normal and cancerous tissues for age- and gender-controlled subgroups. Box plots indicate the median and upper/lower quartiles, with individual data points representing outlier values greater than 1.5x the interquartile range above or below the upper or lower quartile, respectively.

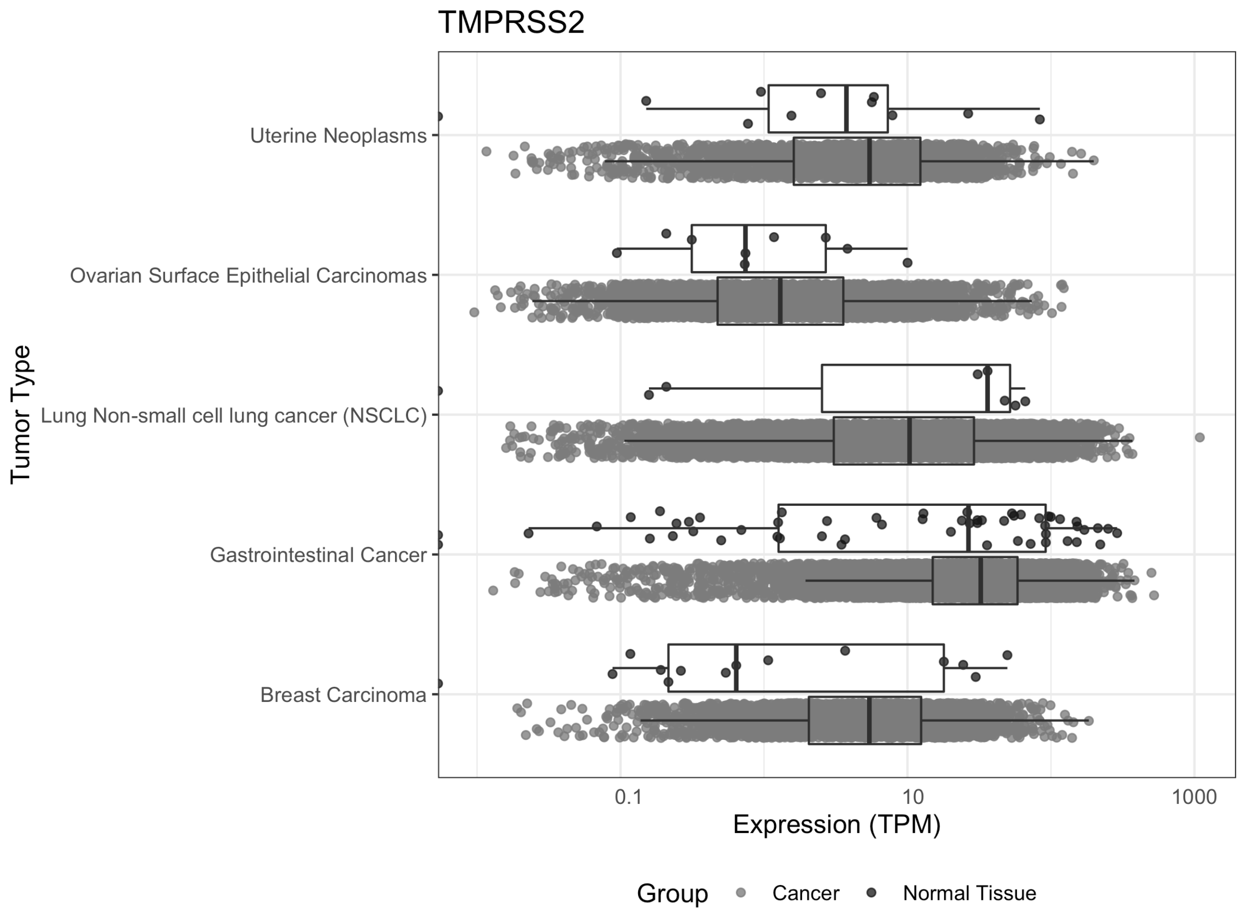

Supplemental Figure 5: TMPRSS2 gene expression in tissue type-matched normal and cancerous tissues. Box plots indicate the median and upper/lower quartiles, with individual data points above/below the whiskers representing outlier values greater than 1.5x the interquartile range above or below the upper or lower quartile, respectively.

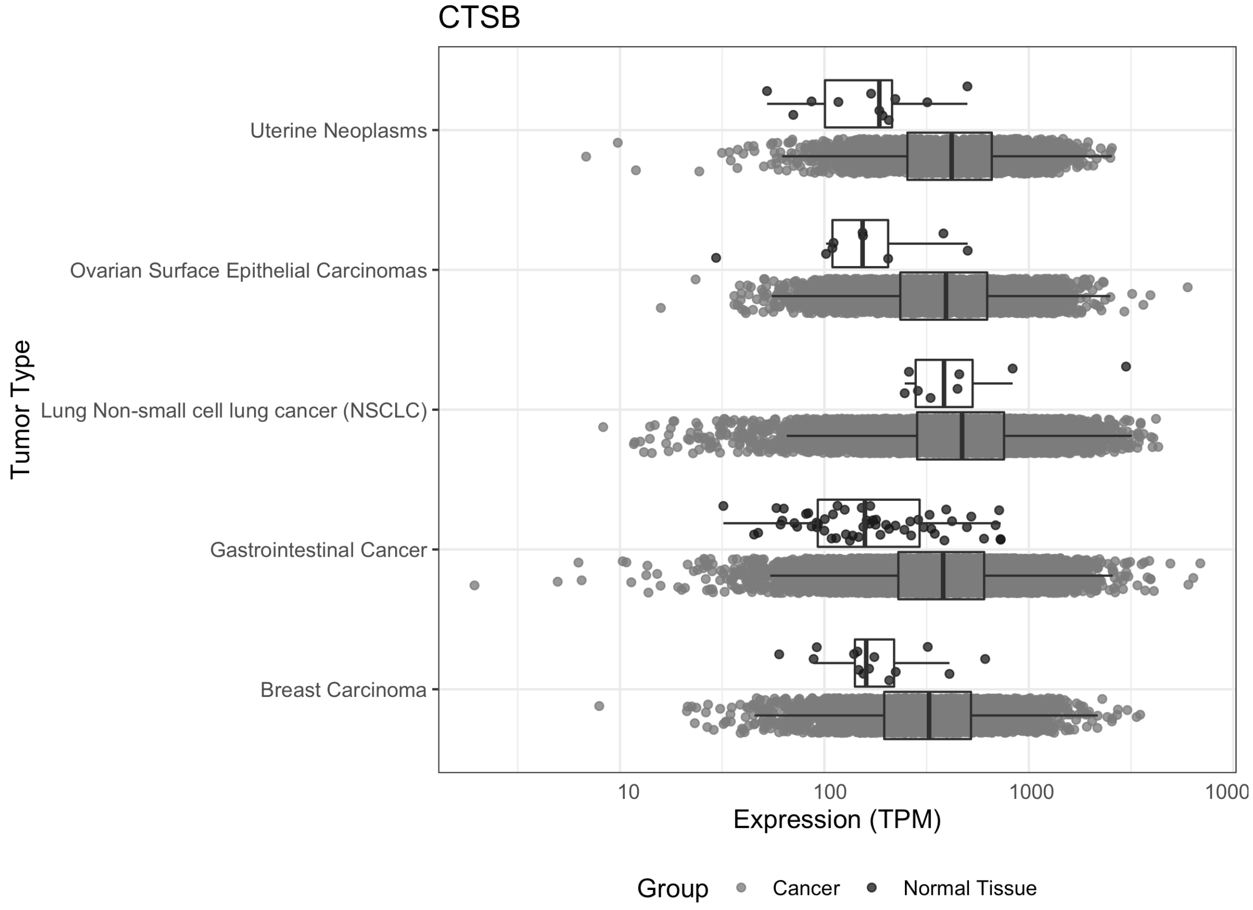

Supplemental Figure 6: CTSB gene expression in tissue type-matched normal and cancerous tissues. Box plots indicate the median and upper/lower quartiles, with individual data points above/below the whiskers representing outlier values greater than 1.5x the interquartile range above or below the upper or lower quartile, respectively.

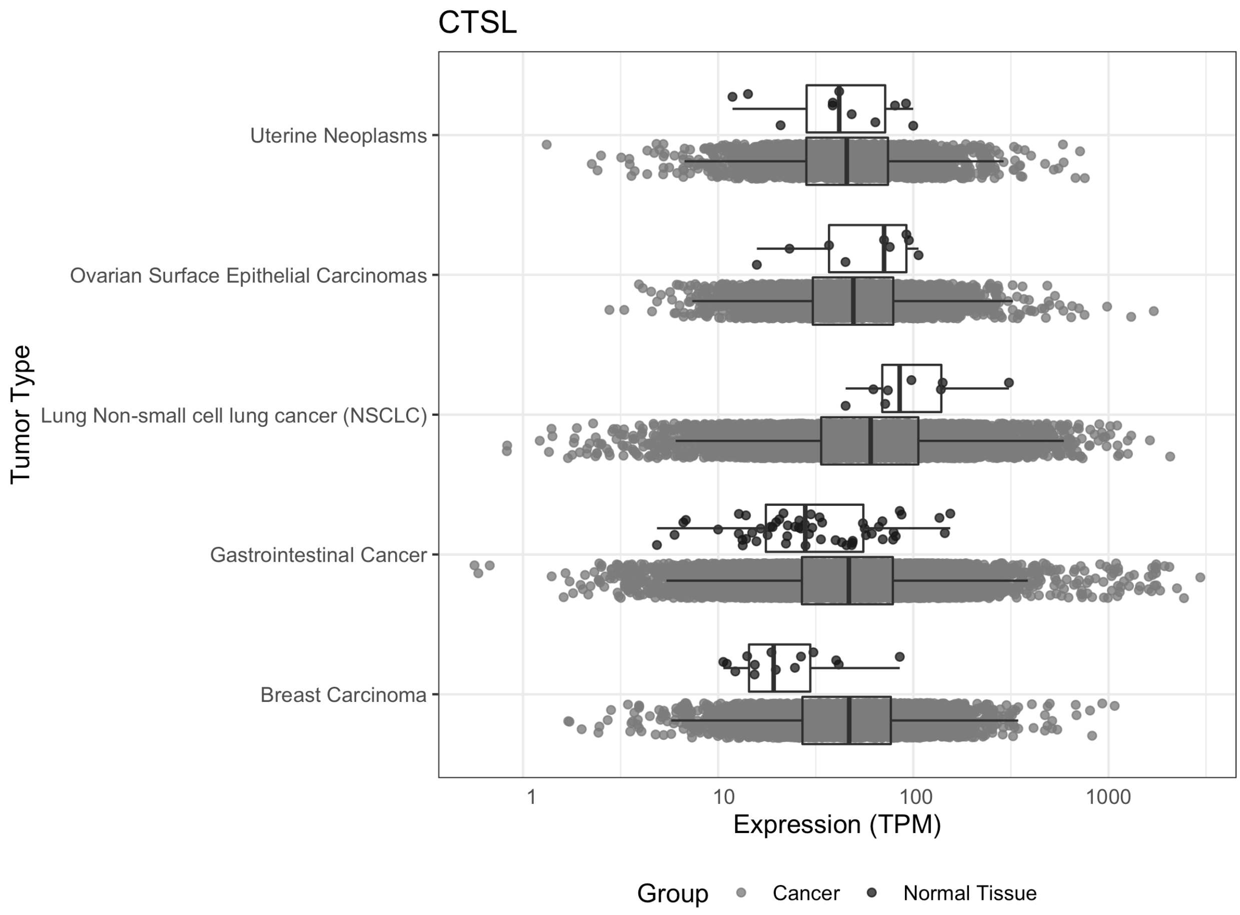

Supplemental Figure 7: CTSL gene expression in tissue type-matched normal and cancerous tissues. Box plots indicate the median and upper/lower quartiles, with individual data points above/below the whiskers representing outlier values greater than 1.5x the interquartile range above or below the upper or lower quartile, respectively.

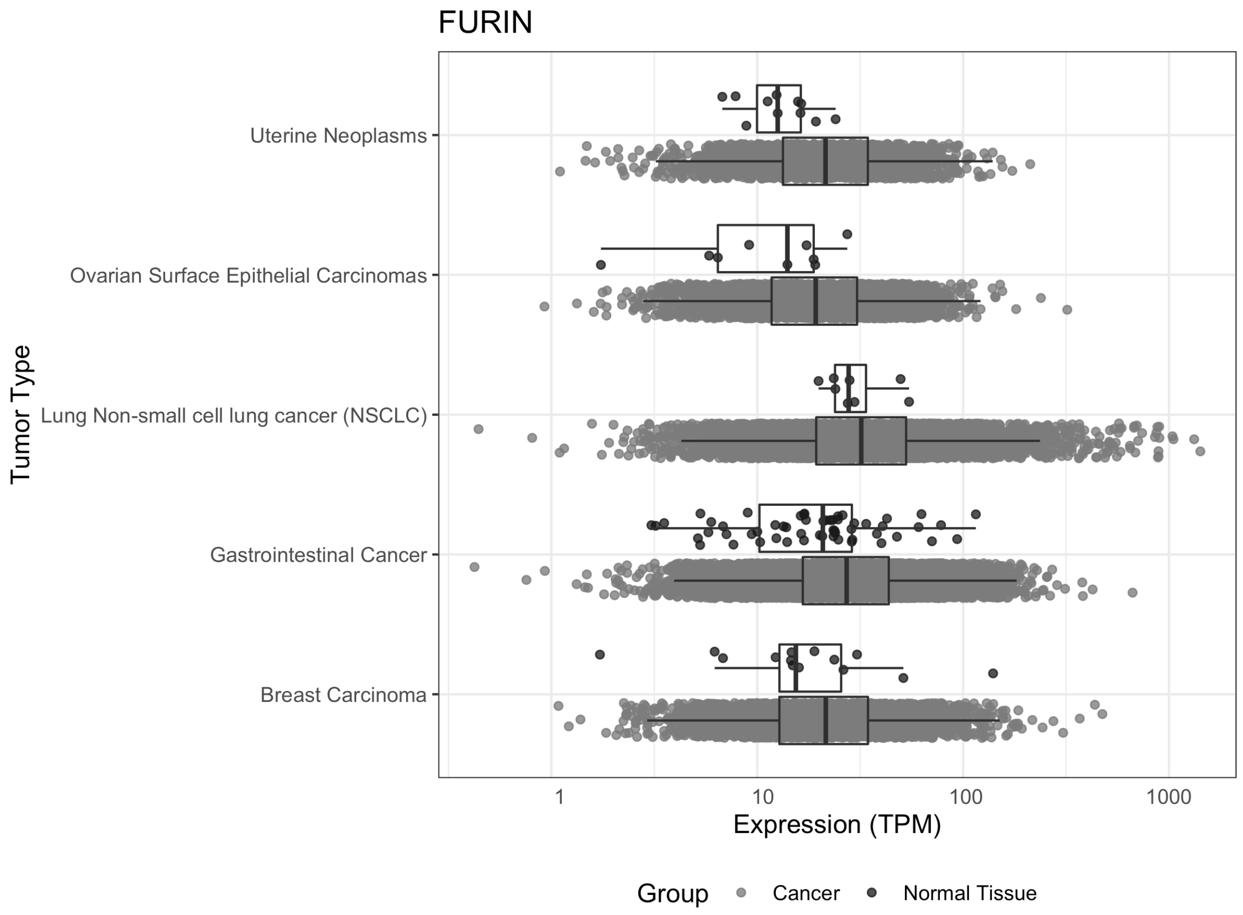

Supplemental Figure 8: FURIN gene expression in tissue type-matched normal and cancerous tissues. Box plots indicate the median and upper/lower quartiles, with individual data points above/below the whiskers representing outlier values greater than 1.5x the interquartile range above or below the upper or lower quartile, respectively.

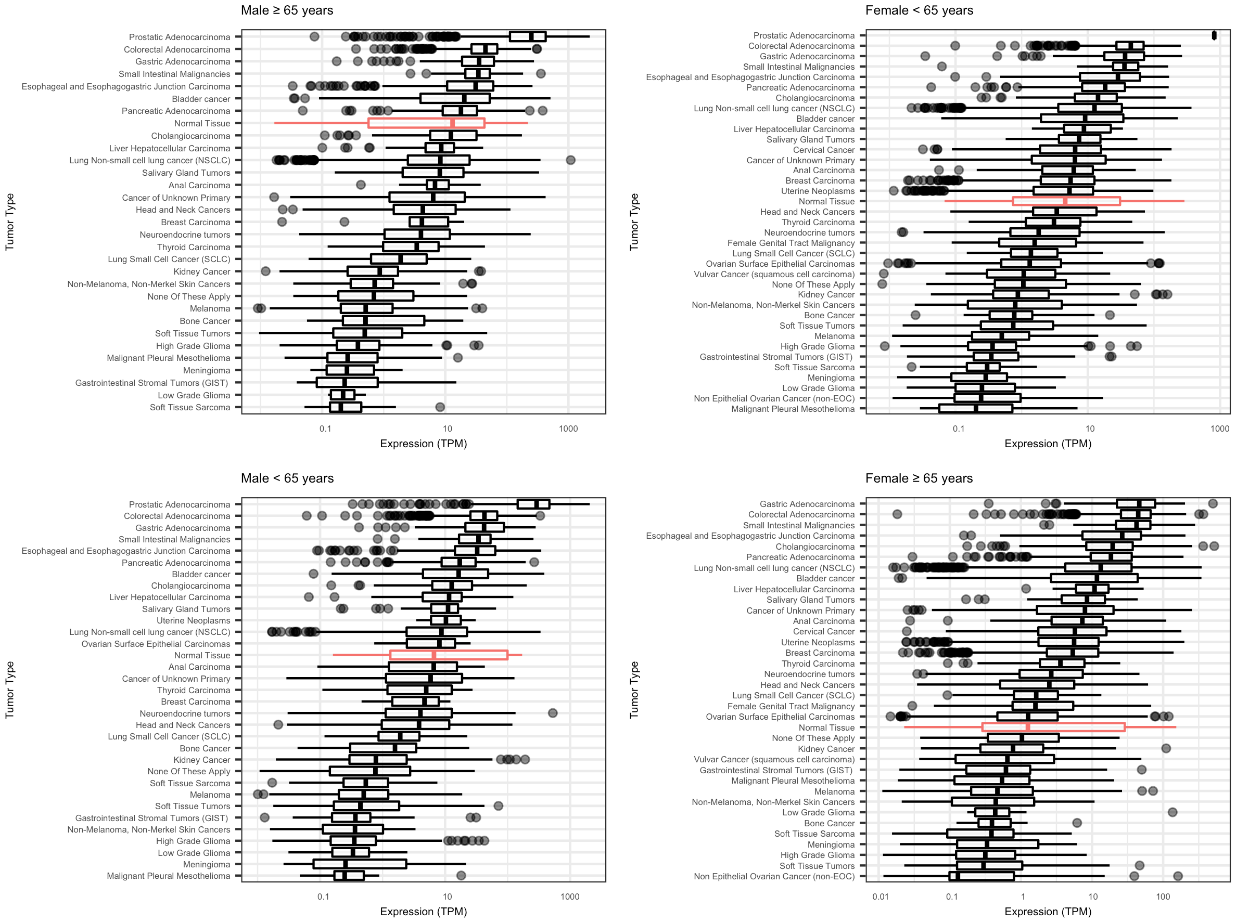

Supplementary Figure 9: TMPRSS gene expression in normal and cancerous tissues for age- and gender-controlled subgroups. Box plots indicate the median and upper/lower quartiles, with individual data points representing outlier values greater than 1.5x the interquartile range above or below the upper or lower quartile, respectively.

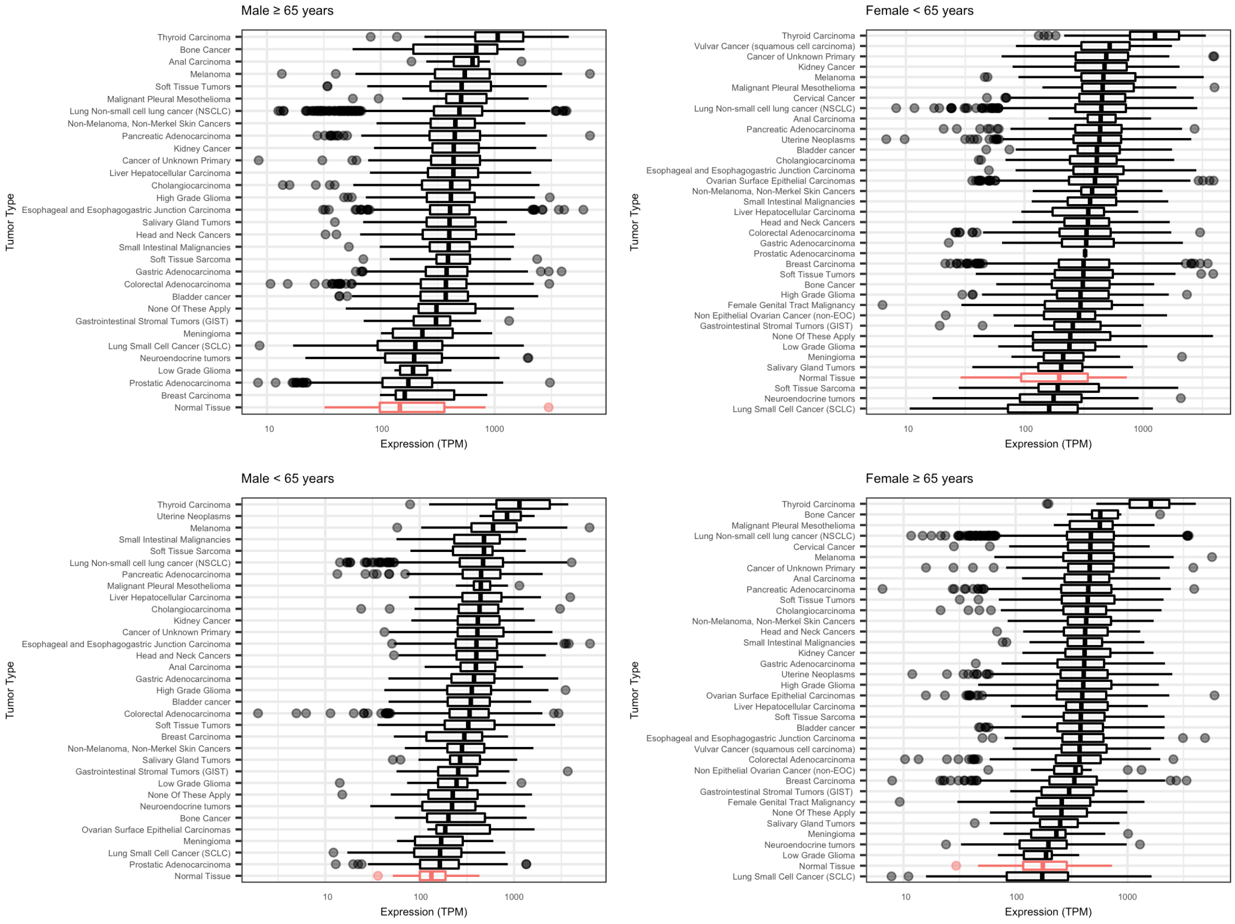

Supplementary Figure 10: CTSB gene expression in normal and cancerous tissues for age- and gender-controlled subgroups. Box plots indicate the median and upper/lower quartiles, with individual data points representing outlier values greater than 1.5x the interquartile range above or below the upper or lower quartile, respectively.

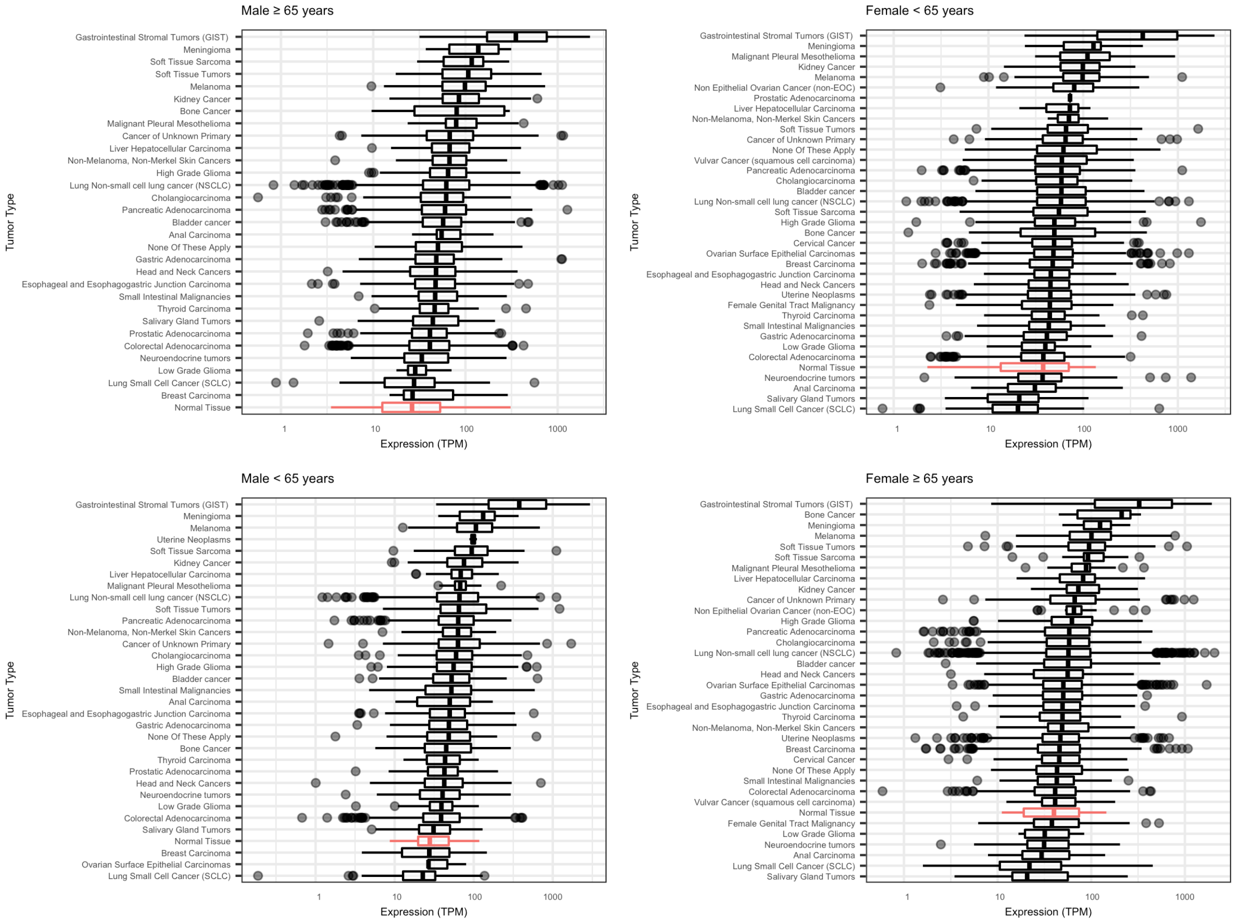

Supplementary Figure 11: CTSL gene expression in normal and cancerous tissues for age- and gender-controlled subgroups. Box plots indicate the median and upper/lower quartiles, with individual data points representing outlier values greater than 1.5x the interquartile range above or below the upper or lower quartile, respectively.

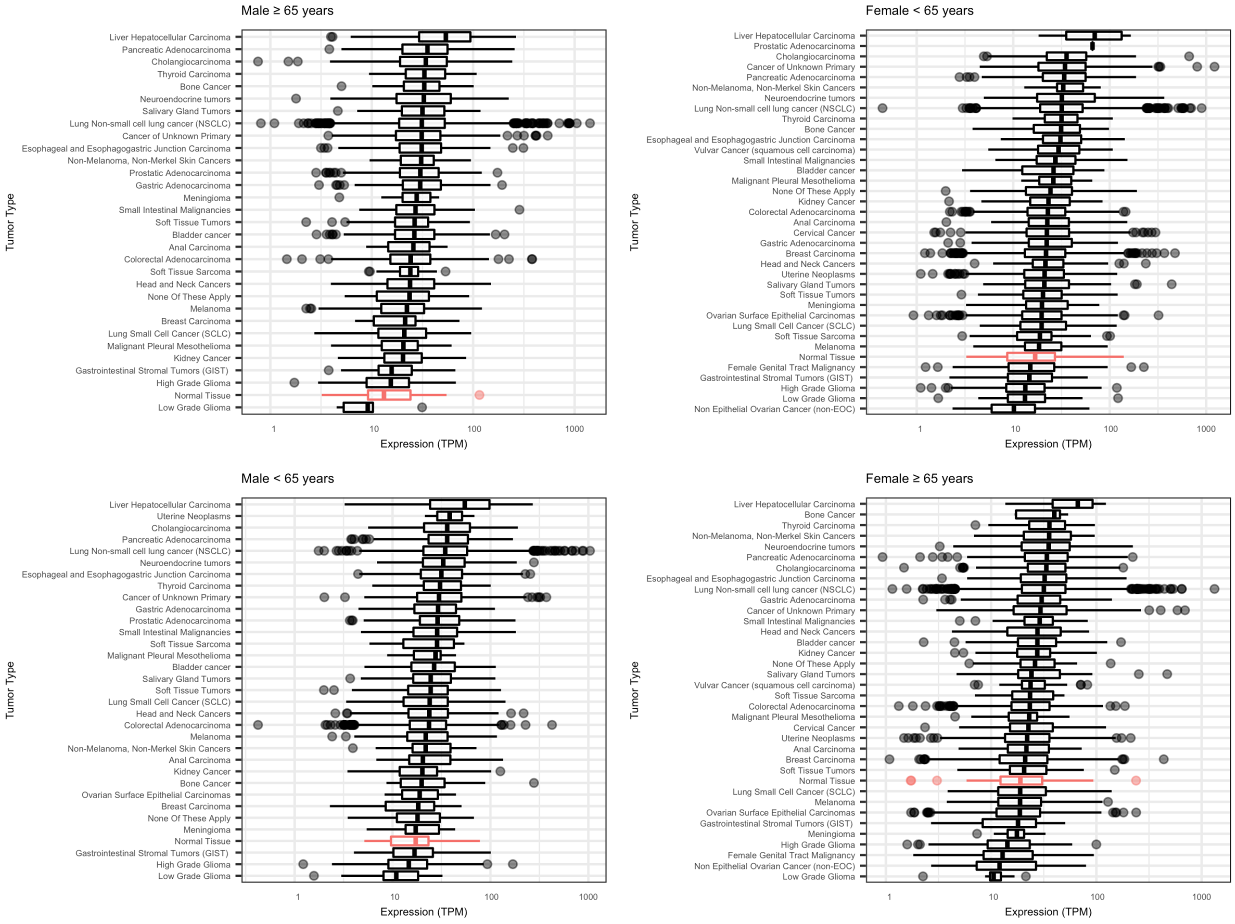

Supplementary Figure 12: FURIN gene expression in normal and cancerous tissues for age- and gender-controlled subgroups. Box plots indicate the median and upper/lower quartiles, with individual data points representing outlier values greater than 1.5x the interquartile range above or below the upper or lower quartile, respectively.
